# Association between rumen microbiota and marbling grade in Hu sheep

**DOI:** 10.3389/fmicb.2022.978263

**Published:** 2022-09-21

**Authors:** Jianghui Wang, Yukun Zhang, Xiaojuan Wang, Fadi Li, Deyin Zhang, Xiaolong Li, Yuan Zhao, Liming Zhao, Dan Xu, Jiangbo Cheng, Wenxin Li, Changchun Lin, Xiaobin Yang, Rui Zhai, Xiwen Zeng, Panpan Cui, Zongwu Ma, Jia Liu, Xiaoxue Zhang, Weimin Wang

**Affiliations:** ^1^College of Animal Science and Technology, Gansu Agricultural University, Lanzhou, China; ^2^The State Key Laboratory of Grassland Agro-Ecosystems, College of Pastoral Agriculture Science and Technology, Lanzhou University, Lanzhou, China

**Keywords:** 16S rRNA, Hu sheep, marbling score, rumen microbiota, muscle chemical composition

## Abstract

The marbling fat regulates the flavor of mutton and measures the fat density in the loin eye and is the most important parameter of carcass grading. The objective of this study was to explore the relationship of rumen microbiota and mutton marbling grade. One hundred and eighty-seven feedlot-finished Hu male lambs (Age: 180 day; Final BW: 46.32 ± 6.03 kg) were slaughtered, and ruminal contents and marbling grade were collected. Ruminal microbial DNA extraction and 16S rRNA gene sequencing was performed to investigate microbial composition and to predict microbial metabolic pathways. The animal cohort was then grouped based on marbling grades [low marbling (LM), marbling grade ≤ 1; Medium marbling (MM), 1 < marbling grade ≤ 3; High Marbling (HM), 3 < marbling grade ≤ 5] and intramuscular fat-associated microorganisms were pinpointed using LEfSe and random forest classification model. Intramuscular fat content had significantly differences among the three groups (*P* < 0.05), and was significantly correlated with VFAs profiling. HM sheep showed a higher abundance of one bacterial taxon (*Kandleria*), and two taxa were overrepresented in the MM sheep (*Pseudobutyrivibrio* and *Monoglobus*), respectively. In addition, the main intramuscular fat deposition pathway was found to involve peroxisome proliferator-activated receptor (PPAR) fatty acid synthesis. By studying the effect of the ruminal microbiome on the marbling of sheep, the present study provides insights into the production of high-quality mutton.

## Introduction

China has rich sheep breed resources, where its mutton production ranks first in the world. Mutton possesses an unique flavor that is linked to the intramuscular fat or marbling fat (Li et al., 2020). Marbling is defined as the appearance of visible white flecks or streaks in intramuscular fat (IMF), which can be detected visually between the bundles of muscle (Nguyen et al., 2021). In the beef industry, the marbling grade is an important index to evaluate the quality of beef. Marbling can be assessed by ultrasound imaging or be visually evaluated by an expert (Nguyen et al., 2017; Harris et al., 2018). Marbling affects the color, flavor, tenderness and juiciness of meat (Stewart et al., 2021). Therefore, a higher marbling grade can obtain greater economic benefits. Particularly in Korea and Japan, producers have attempted to increase IMF to enhance the economic value of beef (Sasaki et al., 2009; Kim et al., 2020). Experts use genetic methods to screen related genes and single nucleotide polymorphisms (SNP)(Sasaki et al., 2009; Kwon et al., 2016). In addition, various nutritional methods are also used, such as oil supplements, vitamin A supplementation and high-grain diet feeding (Schmid et al., 2006; Gorocica-Buenfil et al., 2007; Pickworth et al., 2012; Kim et al., 2020). Previous studies have shown that the formation of marbling is affected by many factors, such as genetic, sexual, nutritional and management factors (Nguyen et al., 2021). Fat infiltration within skeletal muscle is known as IMF, which increases with aging (Yoshiko et al., 2017). The marbling is accumulated through IMF cell hyperplasia and hypertrophy (Harris et al., 2018). Fibro/adipogenic progenitor cells (FAPs) are the main reservoir of IMF cells, and IMF cells primarily develop during the fetal and neonatal stages (Harris et al., 2018; Costa et al., 2021). Research has shown that IMF favors the use of glucose for fat synthesis at early ages (Smith and Crouse, 1984). During later phases of growth, IMF cells may continue to grow and use acetate as a carbon source (Nguyen et al., 2021). In addition, recent research has shown that propionic acid is also involved in fat formation (Zhang Y. et al., 2021). As rumen volatile fatty acids (VFAs) can provide a carbon source for the formation of adipose tissue (IMF), the change of rumen microbiota may be related to the formation of marbling. In addition, several studies have shown that rumen biohydrogenating bacteria, including *Prevotellaceae*, *Lachnospiraceae* and *Rikenellaceae*, provide VFAs (acetic, propionic and butyric acids), which can be positively or negatively affect fat deposition (Zhang Y. et al., 2021). Biohydrogenating bacteria *Butyrivibrio* spp., *Megasphaera elsdenii* and *Propionibacterium* spp. have related fatty acid metabolism, which can affect adipocyte differentiation (Kim et al., 2020).

Many studies have investigated the contribution of rumen microorganisms to growth factors and immunity (Berlutti et al., 2011; Ban and Guan, 2021) and the potential effect of bovine rumen microbiota on marbling (Kim et al., 2020). However, research on this subject, the potential effect of rumen microbiota on marbling, has mainly focused on cattle, with few studies being carried out on sheep. Thus, it is important to reveal the characteristics and functions of the Hu sheep rumen microbiota on marbling. This study aims to research the potential effect of the rumen microbiome on IMF deposition or marbling grade in sheep, which is of great significance to promote the production, research and maximally processed of high-quality mutton in China.

## Materials and methods

### Ethics statement

All experiments in this study were ratified by the Animal Welfare and Ethics Committee of Gansu Agricultural University and implemented in compliance with the Food and Drug Supervision and Administration Regulations of the People’s Republic of China (permit number for conducting animal experiments: NO. 2012-2-159).

### Animals

In present study, 187 Hu sheep from Gansu Wuwei Minqin Defu Agriculture Co., Ltd. (Minqin, China). All sheep were fed in a single pen (0.8 × 1.5 × 1.0 m) and provided with adequate pellet feed and water. Commodity feed was purchased from Gansu Sanyangjinyuan Husbandry Co., Ltd., and the dietary information is listed Supplementary Table 1. The acclimation period was 14 days, the pretest period was 10 days and the trial period was 100 days. The Hu sheep included were slaughtered before morning feeding at 6 months of age, with an average weight of 46.32 kg. All sheep were slaughtered under the supervision of a qualified veterinarian (Supplementary Table 7).

### Marbling grade and muscle chemical composition determination

Visual marbling grades were estimated according to the marbling scoring standard formulated by the agricultural industry standard of the People’s Republic of China (NY/T 630—2002 *Lamb and mutton evaluation and grading*).

After slaughter, the longissimus dorsi of the Hu sheep were obtained, placed at 4°C for 12 h, and then stored at –20°C to test the muscle chemical composition. Meat samples were scanned by reflectance spectroscopy (NIRS) using a FoodScan2 Near-Infrared Meat Fast Analyzer (Foss Science Technology and Trading Co., Ltd., Beijing, China). Samples were scanned twice in duplicate re-packing (resulting in six spectra per sample).

Experimental animals were grouped which based on marbling grade. three marbling grade groups are as follows: low marbling (LM) grade ≤ 1; MM, 1 < medium marbling (MM) grade ≤ 3; HM, 3 < high marbling (HM) grade ≤ 5.

### Microbiological and volatile fatty acids analyses

#### 16S rRNA gene sequencing

After slaughter, the rumen contents were collected from all sheep. Rumen fluid was obtained by four layers of cheesecloth filtration and stored at –80° for subsequent microbiological and VFAs analysis. Microbiological DNA extraction was performed using an EasyPure Stool Genomic DNA Kit (EE301-01; TransGen Biotech, Beijing, China), according to the manufacturer’s guidelines. The 16S rRNA V3-V4 regions were amplified using the primers: 341F: 5′-CCTAYGGGRBGCASCAG-3′ and 806R: 5′-GGACTACNNGGGTATCTAAT-3′. The PCR amplification system has been described previously (Zhang Y. K. et al., 2021). A TruSeq^®^ DNA PCR-Free Sample Preparation Kit (Illumina, USA) was used to obtain the sequence libraries (Schloss et al., 2009). The qualified amplicon libraries were sequenced by using paired-end sequencing on the Illumina NovaSeq PE250 platform. FLASH^1^ was used to splice and assemble reads. Fastqc^2^ was used for quality filtering. The detection and removal of chimeras were performed by UCHIME.^3^

#### Taxonomic and diversity analyses

The available plugins within QIIME2 were used to be taxonomic, such as the DADA2 plugin was used to inspect the quality of the sequence and denoise the reads. Then, the abundance table of amplicon sequencing variant (ASV) was determined. The classify-sklearn plugin was used to train the feature classifier. The naïve Bayesian taxonomic classifier was used to annotate the ASV taxonomy. Alpha Diversity Profiling, Abundance Profiling, Interactive Pie Chart analysis, linear discriminant analysis (LDA) effect size (LEfSe) and random forest analysis were formed using the MicrobiomeAnalyst platform.^4^ LEfSe analysis was used to identify microbial biomarkers, using false discovery rate (FDR) values of 0.05 and a LDA threshold score of 2. Tax4Fun software was used to analyze the functional enrichment of bacterial communities. Kyoto Encyclopedia of Genes and Genomes (KEGG) was used to predict microbial function.

#### Volatile fatty acid analysis

We followed previous research methods when treating rumen liquid (Zhang Y. et al., 2021). VFAs were detected by gas chromatography (ThermoFisher Scientific, Shanghai, China), where 1 μL Samples were injected into a DB-FFAP capillary column (15 m × 0.32 mm × 0.25 μm). The samples were run at a split ratio of 50: 1, with a column temperature of 50–220°C (heating rate = 10°C/min). The injector and detector temperatures were both at 240°C. Peak integration was performed using Chromeleon^®^ Software.

### RNA extraction and real-time fluorescence quantification

The mRNA levels of genes in sheep rumen tissue were quantified for high-marbling grade and low-marbling grade groups by using real-time fluorescence quantification (RT-qPCR). Primers were designed by Oligo 7.0 software (Table 1), total RNA extraction was performed using TransZol (TransGen Biotech, Beijing, China). RT-PCR was performed using a cDNA Synthesis Kit (Yeasen Biotechnology, Shanghai, China), according to the manufacturer’s instructions. RT-qPCR was performed using a Roche LightCycler 480 (Roche Applied Science) and SYBR Green assay (Yeasen Biotechnology, Shanghai, China). The reaction volume was 20 μL, containing 6.4 μL of sterile water, 2 μL of cDNA (100 ng/μL), 0.8 μL of each primer and 10 μL of 2 μL qPCR SYBR Green Master Mix (Yeasen Biotechnology, Shanghai, China). The qPCR conditions were as described in our previous study (Wang et al., 2021). Each gene underwent four technical replicates. The gene expression levels were normalized to that of *ACTB* to determine relative gene expression by using the 2^–ΔΔCt^ value method (Zhang et al., 2019).

**TABLE 1:** Primer pairs designed for target genes.

Primer name	Primer sequence (5′-3′)	GenBank accession number	Annealing temperature (°C)	Size (bp)
*ACADL*-F	ATTGGCAAGATCCATAAGTGA	XM_027965150	51	183
*ACADL*-R	TACTAAAATGATGCTGGCAGT			
*ALDH2*-F	AGGAGATCAGCCACAAGACCA	XM_004017409	58	180
*ALDH2*-R	CACTCTGAAAGCCAGTAGCAGT			
*FGFRL1*-F	CTGAGAGTAAGATGCCTTCCAC	XM_027971341	58	131
*FGFRL1*-R	TCCAAAGGCATTACATGGTGA			
*NHE1*-F	GACCCCTTGCTACCTATGTCC	XM_004005085	60	138
*NHE1*-R	CACCACAAGCAACGACGGAA			
*PCCA*-F	CCGTGAAGCATGTTCCTCA	XM_015098256	57	128
*PCCA*-R	CGCAATTTCTCCTCTATTAGCAA			
*PLCB1*-F	ACTTTGAATTTACTGTTCCGCTT	XM_042230393	58	219
*PLCB1*-R	TGGAAGAAACATCATGCCACA			
*PPARD*-F	TGGCAAAATTCTTCCCTCTGGT	XM_042237247	60	112
*PPARD*-R	AGAAGAGCTGCATTCCTCAGT			
*SIX1*-F	ACGCGCATAACCCTTACCCCTC	NM_001174113	63	144
*SIX1*-R	CGGTGTTCTCCCTTTCCTTGGC			
*ACTB*-F	TCCGTGACATCAAGGAGAAGC	NM_001009784	58	267
*ACTB*-R	CCGTGTTGGCGTAGAGGT			

### Statistical analysis

For data related to phenotypes, descriptive statistics (means, SD). The comparison of groups was conducted using SPSS 26.0 software (SPSS, Chicago, IL).^5^ One Way ANOVA and Least—Significant Difference was used to find significant differences among the group. Spearman’s correlation was used to perform correlation analysis by R software. Linear regression fit was performed to determine relations between marbling and fat data sets (*lm4* in R). And the Linear regression fit model is as follows:


y=i⁢j⁢kμ+m+ib+jn+kei⁢j⁢k

*y*_*ijk*_ is the IMF content, μ is the average value, *m*_*i*_ is the marbling grade (*i* = 0,1,2,3,4,5), *b*_*j*_ is the birthplace (*j* = PD, RL, YS, ZS), *n*_*k*_ is the time (*k* = 2019, 2020), and *e*_*ijk*_ was the residual with a distribution assumption N(0, σ^2^).

## Results

### Marbling grade

**FIGURE 1:**
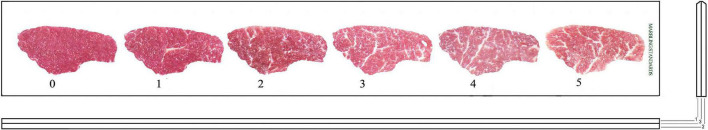
Representative images used for marble grading scale.

Based on the marbling scoring standard formulated by the agricultural industry standard of the People’s Republic of China, members of our team optimized and specified the marbling scoring standard for mutton (shown in Figure 1). 187 Hu sheep were divided into 3 groups based on marbling grade (LM, marbling grade ≤ 1; MM, 1 < marbling grade ≤ 3; HM, 3 < marbling grade ≤ 5), the HM, MM, LM sample sizes were 27, 101, 60, respectively; the average initial body weight were 20.91, 18.89, 17.20 kg, respectively; the average final body weight were 49.17, 46.1, 43.7 kg, respectively (shown in Supplementary Table 7).

### The different analysis with the muscle chemical composition among the marbling grade groups

The difference of the muscle chemical composition among the marbling grade groups were showed that the fat of HM was significantly higher than LM and MM (*P* < 0.05; Table 2), indicating that it is possible for marbling grades to predict IMF content in sheep. While, there are no significant differences in moisture, salt, protein and collagen among groups.

**TABLE 2:** The different analysis with the muscle chemical composition of meat and marbling grade.

Item	Marbling grade groups			SEM	*P*-value
	LM	MM	HM		
Fat (%)	3.69^b^	3.56^b^	4.40^a^	0.17	0.003
Moisture (%)	71.35	71.31	71.08	0.29	0.837
Salt (%)	0.53	0.49	0.54	0.03	0.434
Protein (%)	22.28	22.26	21.80	0.21	0.328
Collagen (%)	1.27	1.29	1.33	0.06	0.804

LM, marbling grade ≤ 1; MM, 1 < marbling grade ≤ 3; and HM, 3 < marbling grade ≤ 5. *P*-value showed the significant differences among the three groups by used one-way ANOVA analysis. Different letters indicate significant differences (LSD, *P* < 0.05). The same letter indicates no significant difference.

### Comparing the major classified taxa and analyzing the differential rumen microbiota

**FIGURE 2:**
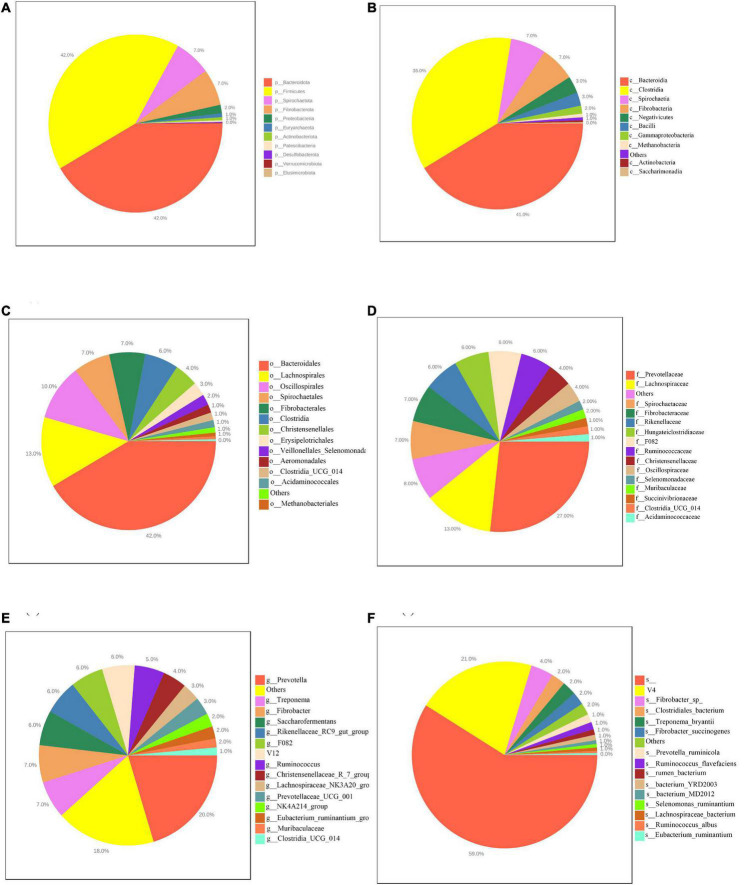
Pie charts showing the average relative abundance of the dominant bacterial phylum **(A)**, class **(B)**, order **(C)**, family **(D)**, genera **(E)** and species **(F)** in the rumen of Hu sheep.

This study obtained 19,449,515 raw reads by 16S rDNA sequencing. After checking for chimeras and picking ASVs, obtained 10,808,820 clean reads with successful retention with an average of 57,801 clean reads per sample. In the rumen, 37 bacterial phyla, 102 bacterial classes, 238 bacterial orders, 412 bacterial families, 893 bacterial genera and 1,324 bacterial species were identified. Figure 2 shows the composition of the rumen microbiota in different level. The results showed that the top 7 sequences were from *Bacteroidota* (42%), *Firmicutes* (42%), *Spirochaetota* (7%), *Fibrobacterota* (7%), *Proteobacteria* (2%), *Euryarchaeota* (1%), *Actinobacteriota* (1%) *Actinobacteriota* (1%), and *Patescibacteria* (0%), at the phylum level, as shown in Figure 2A. at the phylum level, among the major classified taxa *Patescibacteria* was differentially abundant in the LM group (Supplementary Table 2). At the class level, the top 8 sequences were from *Bacteroidia* (41%), *Clostridia* (36%), *Spirochaetia* (7%), *Fibrobacteria* (7%), *Negativicutes* (3%), *Bacilli* (3%), *Gammaproteobacteria* (2%), *Methanobacteria* (1%), *Saccharimonadia* (0%) as shown in Figure 2B. At the class level, among the major classified taxa *Saccharimonadia* was differentially abundant in the LM group (Supplementary Table 3). At the order level, the top 10 sequences were from *Bacteroidales* (42%), *Lachnospirales* (13%), *Oscillospirales* (10%), *Spirochaetales* (7%), *Fibrobacterales* (7%), *Clostridia* (6%), *Christensenellales* (4%), *Erysipelotrichales* (3%), *Veillonellales_Selenomonadales* (2%), *Aeromonadales* (1%), as shown in Figure 2C. At the order level, among the major classified taxa *Lachnospirales* was differentially abundant in the HM group (Supplementary Table 4). At the family level, the top 10 sequences were *Prevotellaceae* (27%), *Lachnospiraceae* (13%), *Spirochaetaceae* (7%), *Fibrobacteraceae* (7%), *Rikenellaceae* (6%), *Hungateiclostridiaceae* (6%), *F082* (6%), *Ruminococcaceae* (6%), *Christensenellaceae* (4%), *Oscillospiraceae* (4%), as shown in Figure 2D. At the family level, among the major classified taxa *Lachnospiraceae* was differentially abundant in the HM group. However, *Prevotellaceae* was differentially abundant in the MM group (Supplementary Table 5). At the genus level, the top 10 sequences were from *Prevotella* (20%), *Treponema* (7%), *Fibrobacter* (7%), *Saccharofermentans* (6%), *Rikenellaceae_RC9_gut_group* (6%), *F082* (6%), *V12* (6%), *Ruminococcus* (5%), *Christensenellaceae_R_7_group* (4%), *Lachnospiraceae_NK3A20_group* (3%), *NK4A214_group* (2%), *Clostridia_UCG-014* (1%) as shown in Figure 2E. At the genus level, among the major classified taxa *Clostridia_UCG-014* was differentially abundant in the HM group, but *NK4A214_group* was differentially abundant in the LM group (Supplementary Table 6). At the species level, the top 10 sequences were from unclassified *species* (59%), V4 (21%), *Fibrobacte* (4%), *Treponema_bryantii* (2%),*Clostridiales_bacterium* (2%), *Fibrobacter_succinogenes* (2%), *Prevotella_ruminicola* (1%), *Ruminococcus_flavefaciens* (1%), *rumen_bacterium* (1%), *bacterium_YRD2003* (1%), as shown in Figure 2F.

**FIGURE 3:**
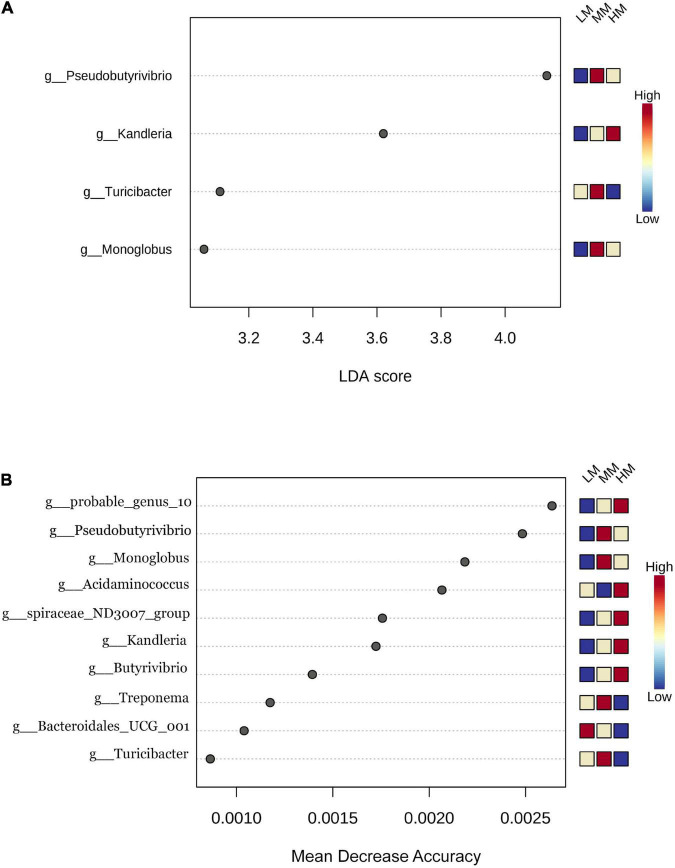
Linear discriminant analysis effect size **(A)**; differentially abundant bacterial taxa defined by LEfSe analysis) computed among low marbling grade (LM), middle marbling grade (MM) and high marbling grade (HM). (LDA > 2, FDR < 0.1) **(B)** the biomarker bacteria classes were identified by applying Random Forest regression analysis of the relative abundance of rumen bacteria.

This study identified differential abundant bacteria composition among the three groups by using LEfSe analysis (linear discriminant analysis score > 2), at the genus level. Meanwhile, 10 different microorganisms were identified when using random forest analysis. Figure 3A shows that *Kandleria, Pseudobutyrivibrio, Turicibacter* and *Monoglobus* were identified as important microbial biomarkers, where only *Kandleria* was enriched in the HM group. Figure 3B shows that probable genus 10, *Acidaminococcus*, *spiraceae ND3007 group*, *Kandleria* and *Butyrivibrio* were enriched in the HM group. *Pseudobutyrivibrio*, *Monoglobus*, *Treponema* and *Turicibacter* were enriched in the MM group. Only *Bacteroidales UCG001* was enriched in the LM group.

### Bacterial function prediction in the rumen of sheep

**FIGURE 4:**
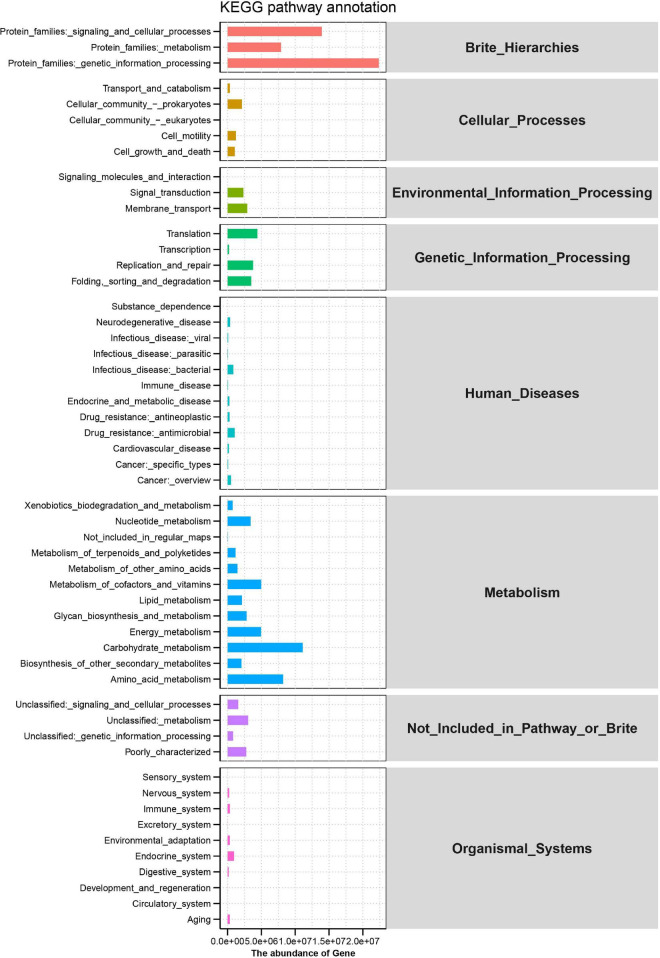
Predicted microbiome function and Kyoto Encyclopedia of Genes (KEGG) pathways in the rumen of Hu sheep.

Rumen microbial functions were predicted by the KEGG pathways analysis. Brite_Hierarchies, Cellular_ Processes, Environmental_Information_Processing, Genetic_ Information_Processing, Human_Diseases, Metabolism, Not_Included_in_Pathway_or_Brite and Organismal_Systems comprises the predominant level 1 KEGG pathways. More detailed functions of microorganisms are shown in Figure 4, where genetic information processing, signaling and cellular processes and metabolism in the protein families had the higher proportion, followed by carbohydrate metabolism, amino acid metabolism, energy metabolism, metabolism of cofactors, and vitamins, nucleotide metabolism, translation, and replication and repair.

### Potential relationship between significant biomarkers and marbling grade

**FIGURE 5:**
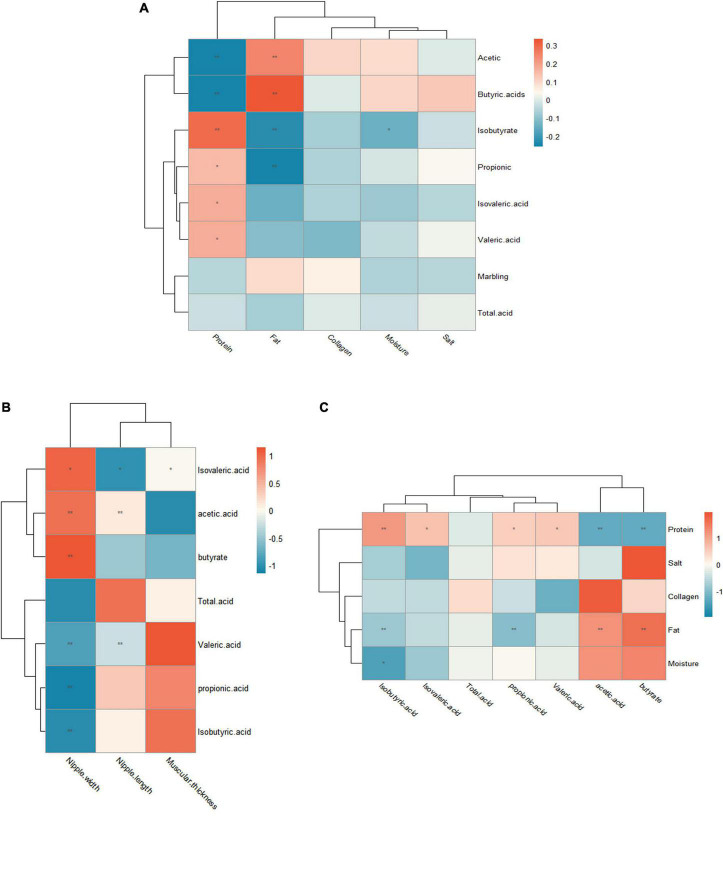
Correlation analysis between significant biomarkers and marbling grade. **(A)** Heat map showing the Spearman’s correlation coefficients among significant biomarkers and marbling grade, VFA. **(B)** Heat map showing the Spearman’s correlation coefficients among muscle chemical composition and VFA. **(C)** Heat map showing the Spearman’s correlation coefficients between VFA and rumen epithelial histomorphology.

There is a positive correlation between significant biomarkers of rumen digesta and marbling grade. *Pseudobutyrivibrio* have positive correlation with marbling grade, whereas *Kandleria*, *Monoglobus* and *Turicibacter* do not have a direct impact on marbling grade. *Kandleria* and *Pseudobutyrivibrio* were closely related to VFAs (Figure 5A).

The correlation between muscle chemical composition and VFAs was further studied. The results showed that there was a positive correlation between fat and butyrate, acetate, whereas propionate and isobutyrate were negatively correlated with fat. In addition, there was a negative correlation between butyrate, acetate and protein. Protein had a positive correlation with valeric acid and isovaleric acid (Figure 5B).

Finally, the correlation between rumen epithelial histomorphology and VFAs was tested. As shown in Figure 5C, there was a significant correlation between rumen morphological development and VFAs. Acetate was positively correlated with ruminal papillae length. Valeric acid and isovaleric acid were significantly negatively correlated with ruminal papillae length. Isovaleric acid, acetate and butyrate were positively correlated with ruminal papillae width. Valeric acid, propionate and isobutyrate were negatively correlated with ruminal papillae width. Isovaleric acid was positively correlated with ruminal muscle thickness.

### Validation of target genes

**FIGURE 6:**
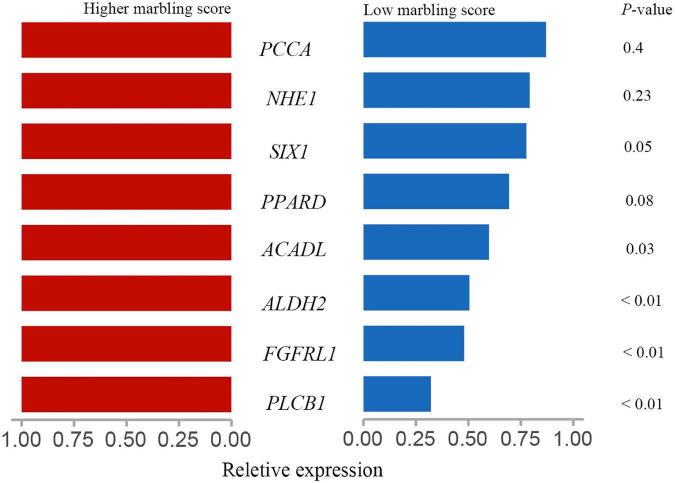
Validation of the target genes in the rumen of Hu sheep. The red indicate the relative expression of target genes in rumen tissue of high marbling grade group; blue indicate the relative expression of target genes in rumen tissue of low marbling grade group. Statistical difference was declared at *P* < 0.05 (*T*-test).

As shown in Figure 6, acyl-CoA dehydrogenase long chain (*ACADL*), aldehyde dehydrogenase 2 family member (*ALDH2*), fibroblast growth factor receptor like 1 (*FGFRL1*), SIX homeobox 1 (*SIX1*), phospholipase C beta 1 (*PLCB1*) expression was significantly higher in the rumen tissue of the LM group than the HM group (*P* > 0.05). There was no significant difference in the expression of solute carrier family 9 member A1 (*NHE1*) or propionyl-CoA carboxylase subunit alpha (*PCCA*) in the rumen tissue of HM and LM groups (*P* > 0.05).

## Discussion

Ruminants use the VFAs and bacterial protein from rumen microorganisms to provide nutrition for themselves. Previous studies have shown that rumen microorganisms are closely related to fat deposition (Zhang Y. et al., 2021). This study found that the marbling grade was significantly associated with IMF content by the different analysis. In addition, this view was verified by linear Fitting (*R*^2^ = 0.1229,*P* < 0.01) (Supplementary Figure 1). This study found a significant correlation between VFAs and IMF content (*P* < 0.05). Therefore, we hypothesized that rumen microorganisms have a potential relationship with marbling grade.

In beef, research has found that the observed ASVs, Chao1 estimates of the HM group were higher significantly than the LM group (Kim et al., 2020). However, in this study, Alpha-Diversity measurements showed no significant difference among the three groups (Supplementary Figure 2), which may be due to the species, grouping and the number of samples. In addition, other studies observed a difference in marbling grades across different residual feed intake (RFI) groups of beef cattle (Ahola et al., 2011). Previous studies have shown that the abundance of *Bacteroidetes* and *Firmicutes* is related to fat deposition (Magne et al., 2020). Our study found that *Firmicutes* were rich in the HM group, while *Bacteroidetes* were rich in the LM and MM groups (Supplementary Table 2). Thus, we are confident that the abundance of *Bacteroidetes* and *Firmicutes* is closely related to IMF.

Among the differentially abundant taxa listed for the HM group, *Kandleria* is considered a lactate producer, where lactate is converted to butyrate by *Megasphaera* spp. (Kumar et al., 2018). The abundance of *Kandleria* increased with the marbling grade. Previous studies have shown that the *Monolobus* is related to host immunity, where the abundance of *Monolobu*s is positively correlated with CD4 + T cell counts and cytokine levels and negatively correlated with the relative numbers of regulatory T cells (Tregs) and T helper (Th17)/Treg ratio (Wang et al., 2022). Our study showed that *Monolobus* can affect IMF deposition, but further research is needed to understand how to affect fat deposition. MM-enriched *Pseudobutyrivibrio* participated in carbohydrate metabolism to produce butyrate (Pidcock et al., 2021). In addition, Correlation analysis shows that *Pseudobutyrivibrio* has made great contributions to the production of VFAs, *Kandleria* plays an important role in the production of Butyrate. Butyrate regulates energy metabolism and increases leptin gene expression (Soliman et al., 2011). Propionate inhibits appetite (Soliman et al., 2011) and is involved in hepatic gluconeogenesis and reduces the expression of enzymes participating in the *de novo* synthesis of fatty acids and cholesterol (Demigné et al., 1995; Chambers et al., 2015). Acetate was also absorbed and reached the systemic circulation and peripheral organs, stimulated the hepatic synthesis of lipids (Gao et al., 2016), and promoted the pancreas to secrete insulin and the gastric mucosa to secrete ghrelin (Perry et al., 2016). The results of our correlation analysis are consistent with the above conclusions (Figure 2), which suggests that butyrate and acetate promote meat with higher marbling formation, while propionate inhibits marbling formation. In summary, it is possible to increase marbling in meat by adjusting the composition of the rumen microbiota.

Prediction of microbiota function indicated that the functions of rumen microorganisms are very complex. Notably, carbohydrate metabolism and amino acid metabolism occupy an important position in the rumen. Carbohydrates are one of the carbon sources of rumen bacteria, especially *Bacteroides* and *Firmicutes*, which can disintegrate complex carbohydrates with the help of digestive enzymes. Amino acid metabolism is one of the nitrogen sources of rumen bacteria. In addition, rumen microorganisms are also involved in lipid metabolism, but the relationship between microbial function and IMF deposition needs to be further explored.

Previous studies indicated that acetate was a substrate for fat differentiation and synthesis, and acetate enhanced the activation of peroxisome proliferator-activated receptor gamma (PPARγ) (Fu et al., 2018). Peroxisome proliferator-activated receptors (PPARs) include three subtypes: PPARα, PPARγ, and PPARβ/δ, where PPARα affects energy homeostasis (Wagner and Wagner, 2020). Some genes are down-stream targets of PPARα and participate in lipid oxidation and metabolism, such as fatty acid degradation and lipoprotein metabolism (Mao et al., 2021). PPARβ/δ regulates fatty acid metabolism (Wagner and Wagner, 2020), and PPARγ enhances fat and glucose metabolism (Wagner and Wagner, 2020). In conclusion, gene regulation plays a key role in lipid deposition.

Butyrate promotes the growth and proliferation of rumen epithelial cells (Luo et al., 2019). However, excessive butyrate can enhance the apoptosis of rumen epithelial cells (Luo et al., 2019). Previous studies have shown that *ACADL*, *ALDH2*, *FGFRL1*, *SIX1* and *PLCB1* are involved in fatty acid degradation, fatty acid metabolism and the PPAR signaling pathway (Bedford et al., 2020; Fang et al., 2021; Shi et al., 2021). In addition, *NHE1* and *PCCA* participate in butyric acid metabolism, phenylalanine metabolism and fatty acid metabolism (Fu et al., 2022; Zhen et al., 2022). Our research found that acetate was used to form IMF lipid by the PPAR signaling pathway. Butyrate and propionate may mainly contribute to the development of the rumen.

## Conclusion

The current study predicted the potential effect of the rumen microbiota to the marbling of lamb. In addition, our research further showed that microbial metabolites (VFAs) regulate IMF deposition through the PPAR signaling pathway and fatty acid metabolism through correlation analysis and qPCR verification. The preliminary study shows that it is possible to develop a strategy for regulating the marbling of meat by microorganisms. However, fat deposition is a complex metabolic process, where host genetic and cell experiments need to be performed to further explore the relationship between IMF deposition and marbling grade.

## Data availability statement

The data presented in the study is deposited in the Sequence Read Archive of NCBI, accession number PRJNA867677.

## Ethics statement

All experiments in this study were ratified by the Animal Welfare and Ethics Committee of Gansu Agricultural University and implemented in compliance with the Food and Drug Supervision and Administration Regulations of the People’s Republic of China (permit number for conducting animal experiments: No. 2012-2-159).

## Author contributions

WW and FL conceived and designed the study. XXZ and XJW revised the manuscript. JW wrote the manuscript. LZ, DX, JC, WL, CL, XY, XWZ, and RZ participated in RNA and DNA extraction. DZ, XL, YKZ, and YZ contributed to the feeding experiment and sample collection. PC, JL, and ZM participated in the measurement of VFA and muscle chemical composition. All authors contributed to the article and approved the submitted version.

## Footnotes

1
http://ccb.jhu.edu/software/FLASH/

2
https://www.bioinformatics.babraham.ac.uk/projects/fastqc/

3
http://www.drive5.com/usearch/manual/uchime_algo.html

4
https://www.microbiomeanalyst.ca/MicrobiomeAnalyst/home.xhtml

5
www.ibm.com/software/analytics/SPSS
