# Supplementary material for: Association between rumen microbiota and marbling grade in Hu sheep
Source: Front Microbiol. 2022 Sep 21;13:978263. doi: 10.3389/fmicb.2022.978263 (PMC9534374; doi:10.3389/fmicb.2022.978263)
Supplement: Supplementary file 1 [file Data_Sheet_1.PDF]

Supplementary Materials

Supplementary Table S1. Diet information for animal experiments

Ingredient Composition(% as fed)		Chemical Composition	
Corn	32.5	Dry matter (DM) [%]	88.78
Corn germ meal	18	Crude protein (CP) [%]	13.09
Corn stalks	12	Digestible energy [MJ/kg]	11.11
Corn hulls	11	Neutral detergent fiber (NDF) [%]	27.08
Corn cob	8	Acid detergent fiber (ADF) [%]	13.99
Soybean meal	4	Crude fiber (CF) [%]	9.78
Cotton meal	5		
Molasses	3.3		
Bentonite	1.5		
Baking soda	1		
Stone powder	0.8		
Expanded Urea	0.7		
Nacl	0.7		
Gypsum powder	1		
Premix	0.5		

Notes: Dry matter (DM; Method 934.01), crude protein (CP; Method 954.01), and crude fiber (CF; Method 962.09) in the feeds were assayed as described by the Association of Official Analytical Chemists (AOAC, 1990). Neutral detergent fiber (NDF) and acid detergent fiber (ADF) were analyzed according to the procedures of Van Soest et al., 1991. Digestible energy was calculated from data provided by the Feed Database of China (the tables of feed composition and nutritive values in China (2016, 15th edition).

Supplementary Table S2. Differences abundance of sheep rumen microbes among marbling score cohorts in the phylum level.

Phylum	Marbling Score Groups[1]			SEM	*P*-Value[2]
	HM	MM	LM		
p__Euryarchaeota	0.20	0.13	0.13	0.04	0.54
p__unclassified	0.13	0.20	0.09	0.07	0.73
p__Actinobacteriota	0.35	0.34	0.28	0.06	0.90
p__Bacteroidota	36.07	41.09	40.00	1.51	0.13
p__Desulfobacterota	0.17	0.22	0.17	0.02	0.28
p__Fibrobacterota	5.61	6.40	6.45	0.62	0.70
p__Firmicutes	47.20	42.30	43.32	1.56	0.10
p__Patescibacteria	0.23^b	0.23^b	0.34^a	0.03	0.03
p__Proteobacteria	2.82	1.49	2.19	0.61	0.18
p__Spirochaetota	6.76	7.28	6.76	0.56	0.70
p__Euryarchaeota	0.20	0.13	0.13	0.04	0.54

Note: LM: marbling score ≤1. MM: 1< marbling score≤ 3. HM: 3< marbling score ≤5. [1]The mean represent the average value of relative abundance (%) of rumen bacterial phyla. [2] One-way ANOVA analysis was performed to determine *P* value. Different letters indicate significant differences (LSD, $P < 0.05$). The same letter indicates no significant difference

Supplementary Table S3. Differences abundance of sheep rumen microbes among marbling score cohorts in the class level.

Class	Marbling Score Groups[1]			SEM	*P*-Value[2]
	HM	MM	LM		
c__Methanobacteria	0.20	0.13	0.13	0.04	0.538
c__Bacteroidia	36.07	41.09	39.99	4.53	0.131
c__Fibrobacteria	5.61	6.40	6.45	1.87	0.701
c__Bacilli	3.37	3.01	3.37	0.98	0.465
c__Clostridia	40.25	35.66	36.57	4.28	0.101
c__Negativicutes	3.59	3.62	3.38	1.30	0.885
c__Saccharimonadia	0.22^{b}	0.23^{ab}	0.34^{a}	0.10	0.044
c__Actinobacteria	0.10	0.16	0.06	0.08	0.742
c__Gammaproteobacteria	2.57	1.26	1.97	1.81	0.176
c__Spirochaetia	6.76	7.27	6.76	1.68	0.702

Note: LM: marbling score ≤1. MM: 1< marbling score≤ 3. HM: 3< marbling score ≤5. [1]The mean represent the average value of relative abundance (%) of rumen bacterial class. [2] One-way ANOVA analysis was performed to determine *P* value. Different letters indicate significant differences (LSD, $P < 0.05$). The same letter indicates no significant difference

Supplementary Table S4. Differences abundance of sheep rumen microbes among marbling score cohorts in the order level.

Order	Marbling Score Groups[1]			SEM	*P*-Value[2]
	HM	MM	LM		
o__Bacteroidales	36.03	41.06	39.96	4.53	0.13
o__Fibrobacterales	5.61	6.40	6.45	1.87	0.701
o__Erysipelotrichales	2.66	2.28	2.52	0.89	0.498
o__Christensenellales	4.94	4.17	4.11	1.39	0.465
o__Clostridia	5.88	5.69	6.02	1.27	0.803
o__Clostridia_UCG-014	1.84	1.38	1.67	0.58	0.098
o__Lachnospirales	16.58^{a}	13.67^{b}	13.19^{b}	2.20	0.007
o__Oscillospirales	10.08	9.93	10.74	1.44	0.327
o__Veillonellales-Selenomonadales	2.71	2.78	2.56	1.11	0.888
o__Spirochaetales	6.76	7.27	6.76	1.68	0.702

Note: LM: marbling score ≤1. MM: 1< marbling score≤ 3. HM: 3< marbling score ≤5. [1]The mean represent the average value of relative abundance (%) of rumen bacterial order. [2] One-way ANOVA analysis was performed to determine *P* value. Different letters indicate significant differences (LSD, $P < 0.05$). The same letter indicates no significant difference

Supplementary Table S5. Differences abundance of sheep rumen microbes among marbling score cohorts in the family level.

Family	Marbling Score Groups[1]			SEM	*P*-Value[2]
	HM	MM	LM		
f__F082	6.08	5.38	5.57	5.68	0.444
f__Prevotellaceae	19.92[b]	26.56[a]	24.71[ab]	23.73	0.033
f__Rikenellaceae	7.65	6.91	7.46	7.34	0.543
f__Fibrobacteraceae	5.61	6.40	6.45	6.16	0.701
f__Christensenellaceae	4.94	4.17	4.11	4.41	0.465
f__Hungateiclostridiaceae	5.88	5.69	6.02	5.86	0.803
f__Lachnospiraceae	16.21[a]	13.37[b]	12.82[b]	14.13	0.007
f__Oscillospiraceae	3.71	3.46	3.89	3.69	0.274
f__Ruminococcaceae	5.02	5.32	5.58	5.31	0.531
f__Spirochaetaceae	6.76	7.27	6.76	6.93	0.702

Note: LM: marbling score ≤1. MM: 1< marbling score≤ 3. HM: 3< marbling score ≤5. [1]The mean represent the average value of relative abundance (%) of rumen bacterial family. [2] One-way ANOVA analysis was performed to determine *P* value. Different letters indicate significant differences (LSD, $P < 0.05$). The same letter indicates no significant difference

Supplementary Table S6. Differences abundance of sheep rumen microbes among marbling score cohorts in the genus level.

genus	Marbling Score Groups[1]			SEM	*P*-Value[2]
	HM	MM	LM		
g__F082	6.08	5.38	5.57	0.42	0.44
g__Muribaculaceae	1.31	1.21	1.11	0.12	0.60
g__Prevotella	15.33	19.54	18.48	1.24	0.13
g__Prevotellaceae_UCG-001	2.09	2.89	2.72	0.28	0.25
g__Rikenellaceae_RC9_gut_group	7.59	6.84	7.40	0.52	0.53
g__Fibrobacter	5.61	6.40	6.45	0.62	0.70
f__Lachnospiraceae;g__unclassfied	1.18	1.37	1.39	0.14	0.73
g__UCG-004	4.87	4.09	4.02	0.46	0.45
g__Christensenellaceae_R-7_group	5.87	5.69	6.02	0.42	0.80
g__Saccharofermentans	1.84	1.38	1.67	0.19	0.10
g__Clostridia_UCG-014	3.35[a]	2.85[b]	2.63[b]	0.17	0.02
g__Eubacterium_ruminantium_group	2.11	2.20	2.01	0.15	0.56
g__Lachnospiraceae_ND3007_group	1.06	1.03	0.89	0.12	0.58
g__Lachnospiraceae_NK3A20_group	3.36	2.55	2.97	0.38	0.25
g__NK4A214_group	2.48	2.26	2.77	0.20	0.09
g__Ruminococcus	4.55	4.79	5.04	0.28	0.57
g__Selenomonas	0.80	1.32	1.18	0.23	0.58
g__Succinivibrio	1.15	0.74	1.35	0.35	0.26
g__Treponema	6.57	7.20	6.74	0.56	0.68

Note: LM: marbling score ≤1. MM: 1< marbling score≤ 3. HM: 3< marbling score ≤5. [1]The mean represent the average value of relative abundance (%) of rumen bacterial genus. [2] One-way ANOVA analysis was performed to determine *P* value. Different letters indicate significant differences (LSD, $P < 0.05$). The same letter indicates no significant difference

Supplementary Table S7. Description of the animals during the preliminary trial

group	*n*	age	gender	IBW[1]	FBW[2]
HM	27	180	male	20.91±4.52	49.17±6.09
MM	101	180	male	18.89±3.69	46.10±5.17
LM	60	180	male	17.201±4.13	43.70±6.82

Note: [1]IBW:Initial body weight (mean±SD, Unit: kg); [2]FBW:Final body weight (mean±SD, Unit: kg)

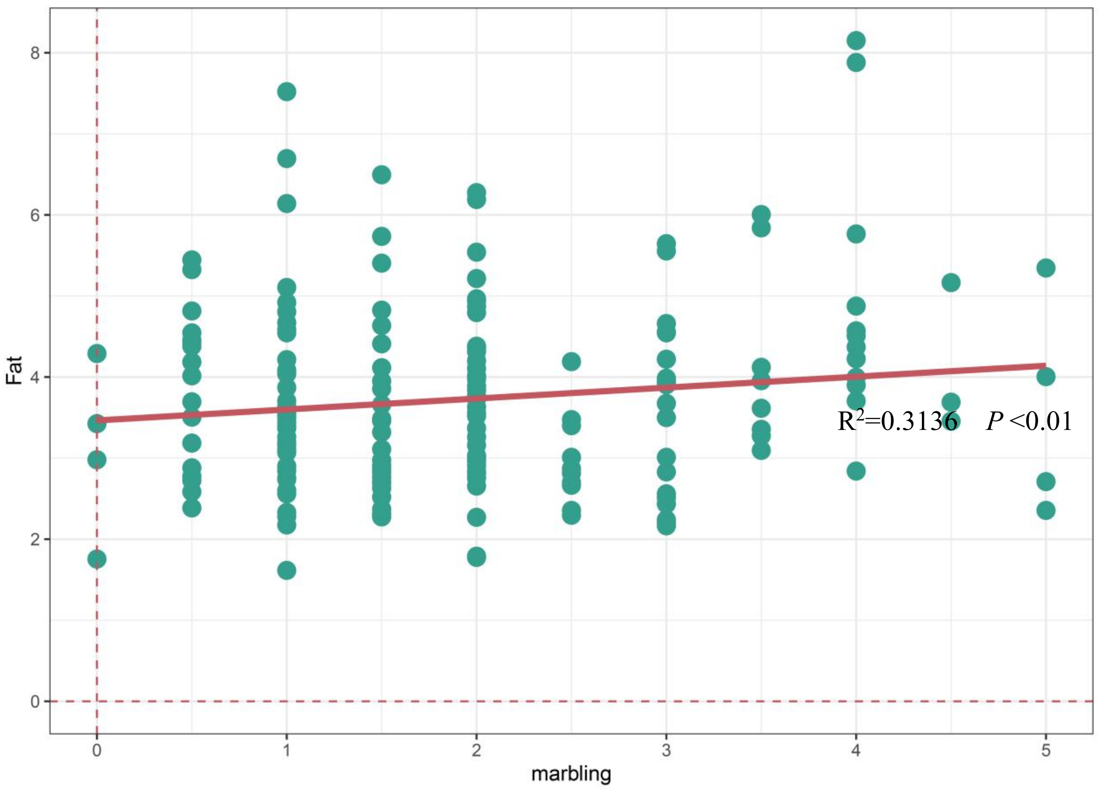

Supplementary Figure S1. Scatter plots were constructed and the linear fit was drawn to analyze the association between marbling and intramuscular fat content (R2=0.3136, P <0.01).

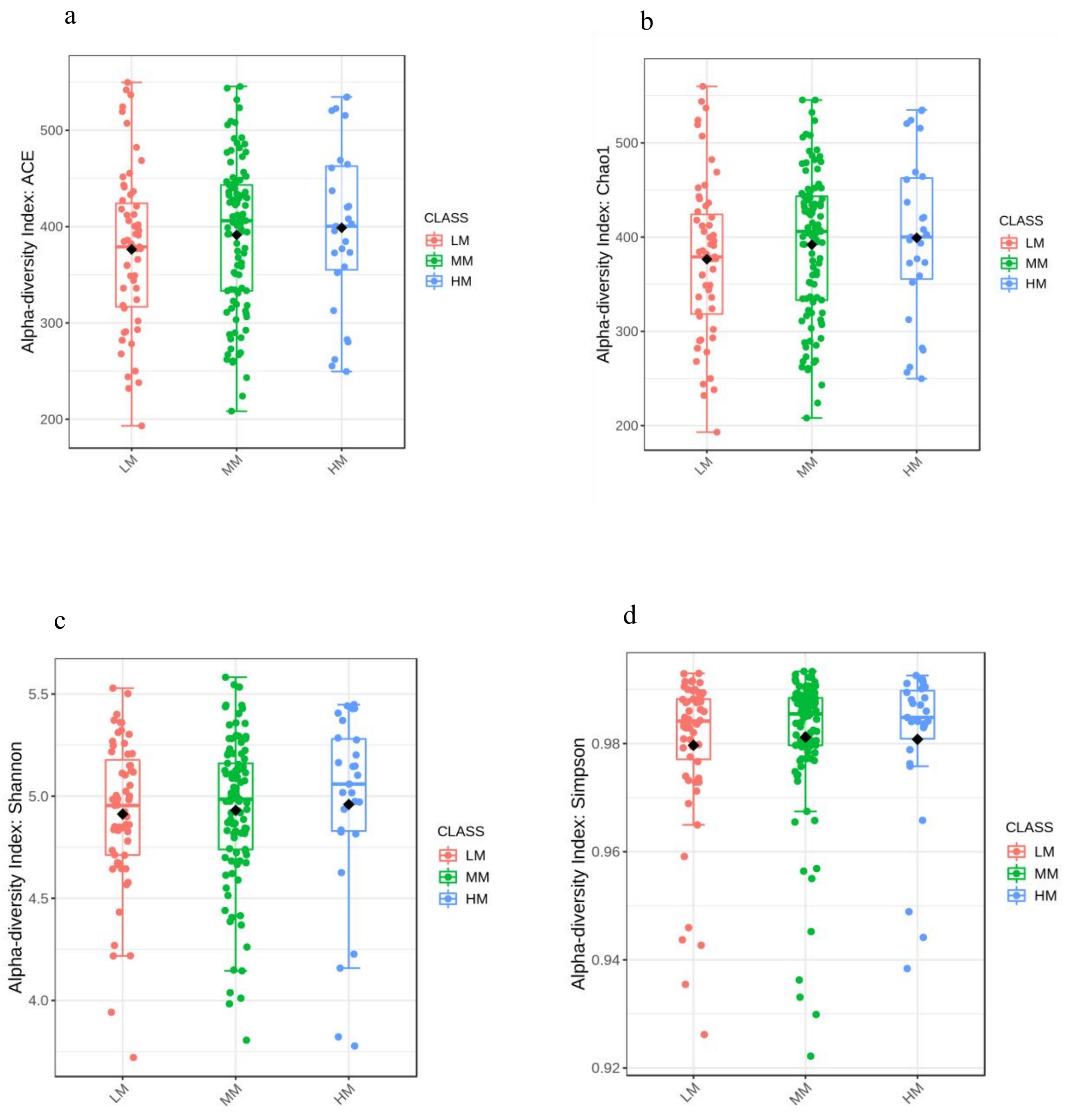

Supplementary Figure S2. Alpha-Diversity measurements of the rumen microbiota in Hu sheep. (a) ACE index (b) Chao1 estimates (c) Shannon diversity (d) Simpson diversity.
